# Coincidence or Causality: Parathyroid Carcinoma in Chronic Kidney Disease—Case Report and Literature Review

**DOI:** 10.3390/diagnostics14111127

**Published:** 2024-05-29

**Authors:** Stefana Catalina Bilha, Anca Matei, Dumitru D. Branisteanu, Laura Claudia Teodoriu, Ioana Hristov, Stefan Bilha, Letitia Leustean, Maria-Christina Ungureanu, Delia Gabriela Apostol Ciobanu, Cristina Preda, Cristian Velicescu

**Affiliations:** 1 Endocrinology Department, “St. Spiridon” Emergency Hospital, “Grigore T. Popa” University of Medicine and Pharmacy, 700115 Iasi, Romania; 2 Department of Medicine, Charles E. Smith College of Medicine, Florida Atlantic University, Boca Raton, FL 33431, USA; 3 Endocrinology Department, Regional Institute of Oncology, 700483 Iasi, Romania; 4 Endocrinology Department, Elytis Hospital Hope, 700010 Iasi, Romania; 5 Department of Nuclear Medicine, Regional Institute of Oncology, 700483 Iasi, Romania; 6 Department of Pathology, “St. Spiridon” Emergency Hospital, “Grigore T. Popa” University of Medicine and Pharmacy Iasi, 700111 Iasi, Romania; 7 Surgery Department, “St. Spiridon” Emergency Hospital, “Grigore T. Popa” University of Medicine and Pharmacy, 700115 Iasi, Romania

**Keywords:** parathyroid carcinoma, secondary hyperparathyroidism, chronic kidney disease

## Abstract

Parathyroid carcinoma (PC) associated with primary hyperparathyroidism (PHPT) has been well investigated in recent years. Data regarding PC evolution in secondary hyperparathyroidism (SHPT) due to chronic kidney disease (CKD) are, however, scarce. Most features that raise the suspicion of PC in PHPT are part of the usual SHPT evolution in CKD, mirroring the natural changes undergone by the parathyroid glands. Therefore, pre-surgically establishing the malignant or benign character of the lesions is cumbersome. We present two cases of PC in end-stage renal disease, one of which was bilateral, diagnosed after total parathyroidectomy in a high-volume parathyroid surgery center. A literature review of the data was also performed. A systematic search of the PubMed/MEDLINE database until January 2024 identified 42 cases of PC associated with SHPT. Understanding the PC features in CKD might improve associated bone and mineral disease management, and reduce the risk of metastasis, parathyromatosis, or recurrence. Irradiation, prolonged immunosuppression, long dialysis vintage, and genotype may predispose to the malignant transformation of chronically stimulated parathyroids. Despite postsurgical diagnosis, favorable outcomes occurred when distant metastases were absent, even without “en bloc” resection. Further research is warranted to delineate specific diagnostic and therapeutic approaches tailored to this particular patient subpopulation.

## 1. Background

Although considered the least common endocrine malignancy (1% of all cases of primary hyperparathyroidism), parathyroid carcinoma (PC) has registered a rise in its incidence in the last 5 decades, partially explained also by the more frequent serum calcium screening or the greater life expectancy [1,2,3]. The cure of PC is largely dependent on surgery, with “en bloc” resection being the gold-standard procedure [1,4]; therefore, preoperative diagnosis is desirable, especially as the decision to perform radical neck surgery is not easy [1,5]. Establishing the diagnosis is challenging, as the clinical spectrum is similar to a benign lesion [2]. The criteria that may differentiate PC from a parathyroid adenoma (PA) or from an atypical parathyroid tumor are the aggressive behavior, severe hypercalcemia and its related complications, and the metastatic potential [2]. These criteria are, however, not always present in PC, and certain PAs (especially those of higher dimensions) can also be accompanied by severe hypercalcemia and/or complications [1,6]. Only histopathology is able to confirm the malignancy, vascular, lymphatic, or perineural invasion being a mandatory feature [2,7].

While the understanding of PC occurrence in primary hyperparathyroidism (PHPT) has evolved in recent years [1,2,3], much less is known about its evolution in secondary hyperparathyroidism (SHPT) due to chronic kidney disease (CKD), hereafter referred to as SHPT.

CKD mineral and bone disorders (CKD-MBD) is an inevitable complication occurring along the natural course of kidney disease, with a significant contribution to the cardiovascular mortality of these patients. Low calcitriol, high fibroblast growth factor 23 (FGF23), chronic hypocalcemia, and hyperphosphatemia lead to SHPT with hyperplastic transformation of the parathyroids, renal osteodystrophy, and vascular calcifications [3,8,9,10]. SHPT is constantly present in end-stage renal disease (ESRD) [10,11]. It often becomes resistant to available medical treatments, raising the need for parathyroidectomy (PTx) [10,11,12]. PC is seldom diagnosed after PTx in SHPT, with only 42 cases reported up to the writing of this manuscript [13,14,15], while the mechanism of parathyroid tumorigenesis is not fully understood [8,9,10,16,17]. Moreover, features that help identify PC in PHPT mimic the natural changes undergone by the parathyroid glands in SHPT (large dimensions, very high parathormone (PTH), high 99mTc-methoxyisobuthylisonitrile (99mTc-MIBI) scintigraphy uptake on scintigraphy, and low expression of vitamin D or calcium-sensing receptor) [11,12,16,18,19]. The management of these patients after diagnosis remains a dilemma, as reintervention poses the risk of parathyromatosis [20]. Nevertheless, reported outcomes differ significantly, even in the absence of “gold standard” surgery [14,15].

We report two cases of PC in ESRD diagnosed postoperatively after total PTx in a high-volume PTx surgery department, among which is a rare finding of bilateral functional PC. Literature data were also reviewed, and key aspects related to the pathogenesis, clinical features, diagnosis, treatment, and outcome of PC in CKD are discussed.

## 2. Case Presentation

### 2.1. Case 1

We herein present the case of a 61-year-old woman diagnosed with ESRD at the age of 44 and undergoing dialysis ever since. Six months prior to her presentation, she was diagnosed with SHPT (PTH = 1048 pg/mL, Table 1) and managed conservatively using calcitriol and paricalcitol without success (Table 1). She complained of severe osteo-articular pain and muscle weakness, while physical examination revealed a cervical anterior painless mass, without any palpable lymph nodes.

**Figure 1:**
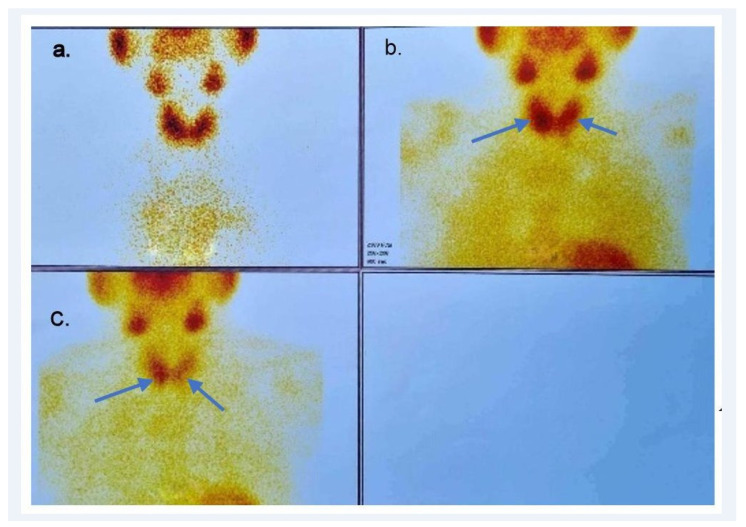
(**a**) 99Tc; (**b**) early 99mTc-methoxyisobuthylisonitrile (99mTc-MIBI); (**c**) delayed 99mTc-MIBI scintigraphy images of the neck: increased radiotracer uptake in the lower third of the right thyroid lobe and posterior to the left thyroid lobe, interpreted as a right inferior and a left inferior parathyroid adenoma, respectively (arrows).

Thyroid ultrasound described a 2.06/1.23 cm hypoechoic mass, located inferior to the right thyroid lobe and also a left 1.04/0.78/1.54 cm posterior subcapsular hypoechoic nodule. These lesions were later confirmed to be PAs on 99mTc-MIBI scintigraphy-single-photon emission computed tomography (99mTc-MIBI-SPECT), which showed increased uptake in the lower third of the right thyroid lobe and posterior to the left thyroid lobe, interpreted as right inferior and left inferior PAs, respectively (Figure 1). Dual-energy X-ray absorptiometry showed a T-score of −2.8 SD for the lumbar spine, −2.6 SD for the femoral neck, and −3.3 SD for the 1/3 radius. Additionally, calcifications were identified in the aortic and mitral valves.

**Figure 2:**
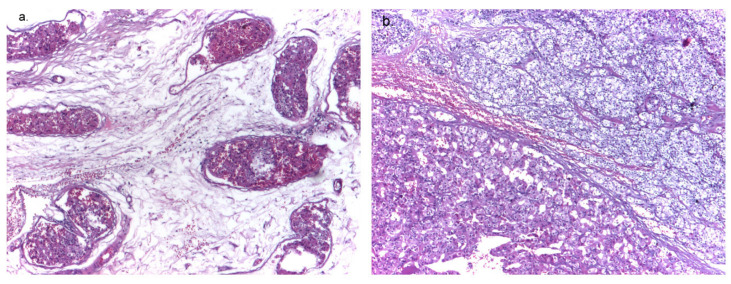
Case 1: left inferior parathyroid gland, hematoxylin–eosin, ×4. (**a**) Vascular tumor embolism in the capsular vessels: deposits of fibrin and parathyroid cells. (**b**) Solid and trabecular architecture with parathyroid main and oxyphil cells.

Due to severe persistent SHPT non-responsive to medical treatment, the patient underwent neck exploration, which revealed enlarged right (largest diameter 3.5 cm) and left inferior (largest diameter 2 cm) parathyroid glands, respectively. Subtotal PTx was performed. The pathology report showed frequent vascular tumor emboli in the surrounding adipose tissue of the left superior and right inferior parathyroid glands. The morphological aspects were consistent with a bilateral parathyroid carcinoma with angioinvasion, stage pT1NxL0V1Pn0 with safety margin excision (Figure 2).

**Figure 3:**
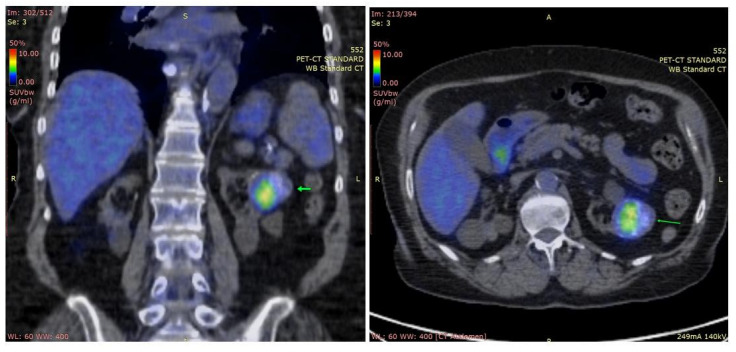
Positron emission tomography: abdominal coronal and axial sections; heterogeneous left renal mass with calcifications, metabolically active (maximum standardized uptake value = 6.3 g/mL; arrows).

PTH levels showed an important early postsurgical drop but tended to increase at 6 and 9 months, despite normal levels of calcium (Table 1). Consequently, a positron emission tomography-computed tomography scan (PET-CT) was performed, showing a metabolically active, heterogeneous, renal mass with calcifications of 37/39/36 mm (Figure 3). Further investigations are needed to distinguish between primary renal lesion and PC metastasis.

### 2.2. Case 2

Our second case is a 50-year-old male patient with CKD due to polycystic kidney disease who had been undergoing dialysis for 20 years. He associated CKD-MBD with severe persistent SHPT refractory to conservatory treatment. The patient complained of severe generalized bone pain and muscle fatigue.

No palpable mass or lymph nodes were found in the cervical area. The patient had very high PTH levels (1911 pg/mL), elevated alkaline phosphatase (ALP = 161 U/L), and mild hypercalcemia (10.4 mg/dL) (Table 2).

He also had secondary osteoporosis with a lumbar T-score of −4 SD, a neck T-score of −3.6 SD, and a 1/3 radius T-score of −3.6 SD for which Denosumab was prescribed.

**Figure 4:**
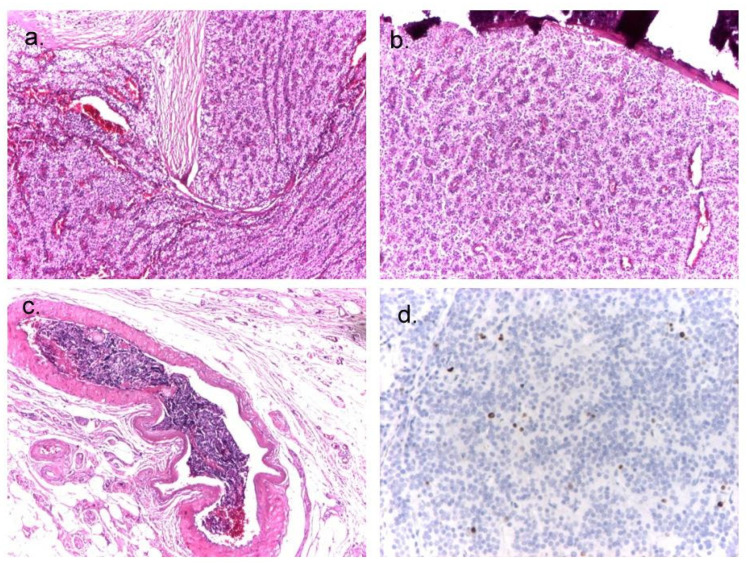
Case 2: right inferior parathyroid gland. (**a**) Capsular infiltration; hematoxylin–eosin, ×4 (on the left of the image); (**b**) acinar architecture with interstitial calcifications; hematoxylin–eosin ×4; (**c**) detail of vascular embolism with deposits of fibrin and parathyroid cells, hematoxylin–eosin, ×10; (**d**) proliferation rate Ki-67 = 5%, ×10.

The patient underwent total PTx. The pathology report was consistent with a low-grade right inferior PC measuring 1.1/0.8/1.5 cm, with a Ki-67 index of 5%, and stage pT1NxL0V1Pn0. Local vascular tumoral emboli were identified, but there was no evidence of nerve or adipose tissue invasion (Figure 4). The safety margin resection was within the surrounding adipose tissue. The other three parathyroid glands showed nodular hyperplasia.

**Figure 5:**
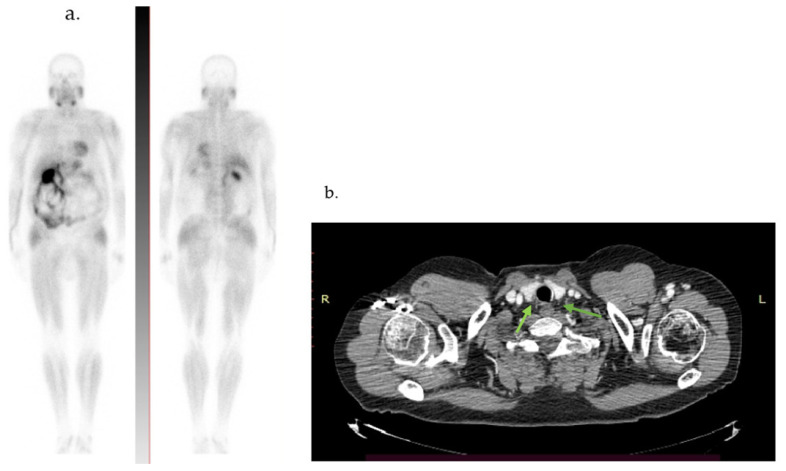
(**a**) 99mTcMIBI whole-body scintigraphy. (**b**) Cervical computed tomography: absence of any signs of recurrence 3 months after surgery (arrows).

Three months after surgery, the whole-body 99mTc-MIBI scintigraphy and computed tomography (CT) scan were negative for any signs of recurrence, ectopic parathyroid lesions, or metastasis (Figure 5). During the 16-month follow-up, the patient’s PTH level decreased to 554 pg/mL, with slight fluctuations due to hypocalcemia (as shown in Table 2). He developed “hungry bones syndrome”, adequately managed with substitutive treatment.

## 3. Literature Review Results

We performed a literature review of published case reports of PC in SHPT to identify similarities regarding the moment of occurrence, diagnosis, type of surgery that was performed, associated risk factors, evolution, and outcomes.

We searched the PubMed/MEDLINE electronic database for articles published from inception to January 2024 using the following keywords: “secondary hyperparathyroidism” AND “chronic kidney disease”/“end-stage renal disease”/“dialysis” AND “parathyroid carcinoma”. Original articles reporting PC associated with SHPT in CKD patients were retained. Relevant references from the selected articles were also searched manually. Systematic reviews reporting PC cases in SHPT were also compared to check for any missed cases.

We identified 42 cases of PC in CKD-MBD patients reported up to the writing of this manuscript, besides the ones presented herein (Table 3). The female-to-male ratio was 1:1, with a mean age of 50.5 years and most of the patients being diagnosed in their fourth and fifth decade of life. Worth mentioning is the presence of risk factors for neoplasia in some of the reports, such as history of neck radiotherapy, immunosuppression, or family history of cancer. Thirty-five out of forty-two patients had hypercalcemia (defined as calcium > 10.5 mg/dL), while mean PTH values were 1379 pg/mL (only clear values reported in pg/mL were used). The mean dialysis vintage was 7.2 years; however, five cases were either kidney transplant recipients (KTR) or had never been on dialysis before being diagnosed with PC. Three patients had multiple synchronous PC as follows: one with four malignant lesions (KTR for 18 years); the other two ESRD patients had two (18 years spent on dialysis) and three (13 years spent on dialysis) malignant parathyroids, respectively. Six PCs were ectopic (two on auto-transplanted parathyroid tissue; four either in the mediastinum or intrathyroidal). A subcategory of patients had mildly elevated PTH values but were resistant to medical treatment and accompanied by hypercalcemia.

Among all PC cases reported up to the writing of this manuscript (Table 3), nine patients underwent neck surgical reintervention and four had parathyromatosis found on this second intervention. Tumor dimension was reported to be between 1 and 5.9 cm, with most being over 2 cm (where available) and seven lesions being over 3 cm. Most patients had local and regional invasion in the thyroid, local vessels, lymph nodes, laryngeal nerve, muscle, and soft tissue, while six patients had distant metastases in the lungs and mediastinum. Finally, out of the 42 patients, 31 had a favorable outcome with a good prognostic at follow-up. Four out of the six patients with distant metastases had a poor prognosis (Table 3).

## 4. Discussion

PC is a rare neoplasm, accounting for less than 0.005% of all malignancies and less than 1% of PHPT [2,3]. PC incidence increased in the last 50 years, from 2 to 10–13 cases per 10 million, likewise because of the increased accessibility to serum calcium measurement [3]. However, improved screening and diagnostic methods may also explain the rather limited dimensions of PC (under 3 cm) and a lack of distant metastases at diagnosis [2]. A recent systematic review performed by McInerney et al. [2] found approximately 2300 PHPT patients reported to have PC.

SHPT is highly prevalent in advanced CKD, with more than 80% of stage 4 and 5 patients suffering from this condition [8]. SHPT starts developing in CKD stage 3, where the risk of having PTH twice above the upper reference limit significantly increases when the eGFR drops below 45 mL/min/1.73 m [9,11]. In a recent study published by Xu et al. [9], the most significant risk factor for developing SHPT was the low eGFR, followed by the presence of diabetes and increased albuminuria. Nonetheless, SHPT is a definitive feature of ESRD, with an incidence of 230 cases/1000 person-years and a more than 90% prevalence [8,9]. Moreover, SHPT prevalence reaches 61% even after kidney transplantation, and hypercalcemia is present in 21.5% of kidney transplant recipients [10]. SHPT is a major comorbidity with adverse health consequences on CKD progression when present in the earlier stages, as well as on the risk of vascular calcifications, major adverse cardiovascular events (MACE), erythropoietin-resistant anemia, fractures, and death [4,9]. In ESRD, the calcium paradox becomes evident: due to increased bone resorption in response to rising PTH levels, calcium shifts from bone to vascular smooth muscle cells, leading to arterial stiffness. Up to 70% of dialysis patients associating with severe SHPT also have moderate to severe coronary artery calcifications [18]. The KDIGO guideline on CKD-MBD in 2017 emphasizes the importance of early recognition and treatment of SHPT due to the associated increased morbidity and mortality [11]. Counteracting the stimuli for PTH increase—such as hypocalcemia, hyperphosphatemia, high phosphate intake, and vitamin D deficiency—are desirable as early as possible. Calcitriol and vitamin D analogues are reserved for stages 4 and 5 with severe and progressive SHPT, while calcimimetics are to be added in CKD 5D, where PTH should optimally be kept between two and nine times the upper normal limit according to KDIGO [11]. Failure of response to medical and pharmacological treatment in CKD G3a-G5D with severe SHPT is an indication for PTX according to the abovementioned guideline [11]. In the early course of SHPT development, the diffuse hyperplasia of parathyroid glands usually responds to a pharmacological approach. If left unrecognized or inappropriately managed, adenomatous-like nodular hyperplasia develops, which is associated with reduced expression of vitamin D and calcium-sensing receptors, and thus being less responsive to vitamin D analogues or calcimimetics [16].

Although (1) KDIGO 2017 [11] established guidelines for regular PTH and calcium screening according to CKD stage, with 1–3 months and 3–6 months intervals, respectively, in G5D, and (2) almost all patients undergoing dialysis develop SHPT, the overall reported testing rate is suboptimal worldwide [8,50,51]. The percentage of testing for PTH values in CKD G5 varies between 36% and 48% [50,51].

Thus, up to 15% of CKD 5D patients become resistant to medical therapy after 10 years of dialysis and need PTx. The need for PTx increases with the duration of dialysis, reaching almost 40% after 20 years of ongoing renal replacement therapy [4].

Although the rate of PTx in dialysis patients has decreased in recent years due to the wider use of calcimimetics, PTx has numerous advantages as it prevents tissue calcifications and bone loss, thereby improving survival and quality of life [4]. Persistently elevated levels of PTH (>800–1000 pg/mL in the absence of hypocalcemia) that fail to respond to a combination of calcimimetics and vitamin D analogues for more than 6–12 months are generally accepted as an indication for PTx [4,52].

In ESRD, PTx decreases all-cause mortality by approximately 30% and cardiovascular mortality by approximately 40%, according to the meta-analysis performed by Apetrii et al. [12,53]. When compared to cinacalcet treatment, PTx is associated with better survival, especially in patients with basal PTH levels ≥ 500 pg/mL or calcium ≥ 10 mg/dL in dialysis patients [54,55]. PTx also increases bone mineral density (BMD) more than cinacalcet does in peritoneal dialysis patients with advanced SHPT [55].

PTx, thus, remains one of the most frequently performed surgeries in ESRD, with an incidence that reaches 30 cases/1000 patients-years in CKD 5D patients having been on RRT for more than 10 years [56].

Of all ESRD patients undergoing PTx, nodular hyperplasia is the most common histopathological result reported in the literature, followed by diffuse hyperplasia, while malignancy is found only in an anecdotic number of cases [14,57]. Preoperative higher MIBI uptake is, generally, associated with a considerably higher prevalence of nodular hyperplasia, higher gland weight, and greater cell proliferation [57,58]. Yokoyama et al. [14] identified 37 PC cases in SHPT reported until 2022. Up to the writing of this manuscript, five more cases have been published (Table 3). Around 3% of all PC cases were identified in dialysis patients, despite the significantly higher PTH concentrations in ESRD-related SHPT [15,43]. Nevertheless, there may be some degree of superposition between SHPT and PHPT with renal involvement, as renal involvement was reported in 32% to 84% of malignant parathyroid tumors [3,59].

Early reports raised the concern of SHPT being a predisposing factor for malignant parathyroid tumor development after PC was diagnosed at necropsy in a patient undergoing dialysis who had received radiotherapy for laryngeal carcinoma [21,59]. Other cases also had risk factors for malignancy, such as the long duration of immunosuppression in KTR.

The female patient in our manuscript also had bilateral PC and had been on dialysis for 17 years before being diagnosed. Taking into account the natural evolution of parathyroid disease in SHPT, from polyclonal expansion manifested as diffuse hyperplasia to monoclonal expansion translated into nodular adenoma, early reports proposed malignant transformation to be the end-phase of this natural evolution after many years of living with ESRD (“multi-step hypothesis” of carcinoma development) [28], contrary to PC-PHPT, where malignant lesions are spontaneous and not evolving from a PA [60].

However, some of the PC-SHPT patients reported in the literature had a shorter history of dialysis; therefore, other risk factors for malignancy should be considered, such as neck irradiation, KTR immunosuppression, or genetic variants.

A recent study outlined the genetic and molecular pathways of parathyroid carcinogenesis [17]. Loss of parafibromin due to mutations in the cell division cycle-73 gene-*CDC73* was identified in up to 75% of familial and sporadic PC cases in PHPT. PC also displays alterations of the phosphatidylinositol-3-kinase (***PI3K***)/***Akt*** and the mammalian target of rapamycin (***mTOR***) signaling pathway, changes in microRNA expression profiles, and gene promoter methylation patterns [17]. However, genetic evaluation was not performed in any of the reported cases of PC-SHPT.

Benign SHPT involves: (1) reduced expression of parathyroid regulating receptors like calcium sensing receptors (CaSRe); (2) activation of epidermal growth factor (EGF)/transforming growth factor alpha (TGF-α) pathways; Cyclooxygenase-2-Prostaglandin E2 pathway (COX-2/PGE2); and mTOR signaling, which promotes parathyroid cell proliferation [16]. Whether these factors might also explain the malignant transformation in SHPT after a long dialysis vintage remains to be explored. Multiple PC in PHPT is extremely rare, even in the presence of the abovementioned genetic alterations, with only a few cases reported [61] compared with SHPT, where five of forty-three cases were synchronous [32,40,55], thus supporting the “multi-step hypothesis”. However, other factors like neck irradiation, or long immunosuppression in KTR, were involved in these cases. Consequently, most probably, carcinogenesis in SHPT has superposable mechanisms.

The diagnosis of PC is usually made after surgery on histopathological analysis. This is not ideal, as inappropriate resection and manipulation of the tumor may lead to parathyromatosis (loco-regional spillage and seeding of malignant parathyroid tissue) and recurrence, as reported in some cases [20]. Thus, precise pre-operation diagnosis and localization are desired, especially as complementary treatment options are limited; further, the prognosis in PC is poor if positive surgical margins or distant metastases are present [1]. In practice, all reported cases of PC on SHPT were diagnosed after surgery (Table 3).

Although the occurrence of PC in PHPT has been extensively discussed, with Karakas et al. [62] proposing a logarithmic equation in order to calculate the preoperative risk of PC in primary disease and a recent review in 2023 [1] proposing a diagnostic algorithm of PC in patients presenting with PHPT, much less is known about in SHPT.

Clinical manifestations of PHPT-related PC include severe bone pain, kidney disease, fatigue, a cervical mass, and neuropsychiatric symptoms [1], all of which may be encountered in benign SHPT in ESRD.

Classically, very high calcium levels above 14–15 mg/dL accompanied by PTH levels more than five times above the upper normal limit, high ALP > 285 IU/L, significantly decreased 1/3 radius BMD or gland diameter > 3 cm should raise the suspicion of PC in PHPT patients [1,15,46,63]. However, these features do not apply to SHPT, where nodular hyperplasia can reach significant enlargement, the serum levels of ALP and PTH are highly elevated, and 1/3 radius BMD is compromised. Only two cases of PC on SHPT reported in the literature had serum calcium > 14 mg/dL (Table 2). However, hypercalcemia was almost unanimously present (Table 3), and our second patient also exhibited mild hypercalcemia.

The use of calcitriol, vitamin D analogues, or calcimimetics in ESRD may limit the rise of PTH values in SHPT, thus explaining the rather lower PTH concentrations in PC, thereby making its preoperatory diagnosis difficult. The diagnosis may further be hindered by the down-regulation of calcium-sensing receptors found in both resistant SHPT and PC, irrespective of etiology [64]. Thus, moderately elevated PTH values but resistant to calcimimetics should raise concern.

Cavalier et al. [65] identified an overproduction of the amino-terminal form of PTH (N-PTH) in PC, measured only by the third-generation PTH assay. Thus, a third/second-generation PTH ratio > 1 may act as a marker for PC in PHPT [65]. However, N-PTH overexpression also occurs in ESRD, where a third/second-generation PTH ratio > 1 may reflect severe, albeit benign parathyroid hyperplasia rather than PC [66] and is, therefore, not useful.

Dual-phase Tc99-MIBI scintigraphy is usually performed in SHPT when surgery is envisaged, to map hyperplastic parathyroid glands [67]. Although Tc99-MIBI uptake on early versus delayed acquisitions does not seem to discriminate between the benign and malignant parathyroid lesions, Zhang et al. [68] showed that PC has a higher retention index (RI) of Tc99-MIBI than benign tumors: when the RI peak of the lesion is >−19%, there is a strong suspicion of PC in patients with PHPT. Adding the size of the parathyroid and the PTH level to form a joint index further improves the diagnostic sensitivity (95% in PC on PHPT) [68]. The higher RI may be explained by the lower expression of the P-glycoprotein and multidrug resistance-associated protein 1 (MRP1), which transport drugs and their metabolites across the cell membrane [19,68]. Whether this may apply to PC in SHPT remains unclear, especially as parathyroid MIBI uptake in SHPT is reduced by the administration of calcitriol and calcium, and MIBI uptake and washout correlate with parameters related to CKD (higher uptake and lower washout with longer dialysis vintage and higher PTH and Ca x P product) [69].

CT provides information on the tumor’s size, local invasion, or metastases (bone, hepatic, mediastinal, or pulmonary) [1], but is not routinely performed before surgery unless there is uncertainty regarding ectopic parathyroid tissue, concurrent thyroid pathology that needs further cervical imaging, or suspicion of malignancy. Magnetic resonance imaging has low sensitivity and specificity in the detection of parathyroid hyperplasia but may be useful in detecting recurrence and metastasis [70]. Combining ultrasound (hypoechoic, heterogeneous, lobulated, ill-defined mass with a thick capsule and a depth/width ratio > 1), CT (infiltration of surrounding tissue, calcifications, malignant lymph nodes, high short-to-long axis ratio), and MIBI imaging (increased uptake, peak RI > −19%) increase sensitivity to 100% for PC localization [1,15,71]. However, this is rarely performed in practice.

In case of suspicion or uncertainty of PC, fine-needle aspiration cytology is to be avoided as it increases the risk for parathyromatosis and has a high rate of false-negative results [15].

PET-CT is usually employed 3 to 6 months after surgery for remnant tissue or secondary lesions assessment. False-positive results may occur with postoperative inflammation or inflammatory lymph nodes [15,72]. A cost-efficient alternative is represented by a whole-body scan with Tc99-MIBI, which was performed on our second patient after surgery.

Thus, PC is generally incidentally discovered after surgery for SHPT. Intraoperative clues that should draw attention according to Radu et al. [15] are increased dimensions over 3 cm (of limited value in SHPT, where all parathyroids tend to be larger, but at the same time, the largest parathyroids of all four were malignant in our both cases), irregular margins, high consistency and dense capsule, adhesion to local structures, and enlarged local lymph nodes. The gold-standard for PC remains the “en bloc” resection, consisting of removal of the parathyroid lesion; surrounding fat; ipsilateral thyroid lobe; and, depending on invasion, the recurrent laryngeal nerve and ipsilateral central neck lymph nodes (prophylactic dissection is not yet endorsed), with safety margins. Capsule rupture should be avoided due to the risk of parathyromatosis [1,73]. However, “en bloc” resection should be performed by an experienced high-volume surgeon and is difficult to perform without a preoperative diagnosis (it is difficult to expand the incision, lack of consent of the patient to remove other important structures such as ipsilateral thyroid lobe or recurrent laryngeal nerve if necessary, and assuming responsibility to perform radical surgery without a definite diagnosis) [5]. This explains why most cases are re-operated using a more radical approach after the initial surgery. Furthermore, a few cases were missed at initial histopathological analysis, with secondary lesions or parathyromatosis becoming evident months to years after initial surgery [9,28,32,36].

Histopathological confirmation of PC needs the presence of unequivocal perineural, lymphatic, or vascular invasion as minimal criteria and can be assisted by biomarkers such as galectin-3 increased expression or loss of nuclear parafibromin immunoreactivity [17,74]. Fibrosis, necrosis, and increased mitotic activity are troublesome but are not necessarily diagnostic of malignancy, as they can be displayed by hyperplastic parathyroids or by atypical PA as well [1,74]. Atypical PA shares characteristics with PC, like increased capsular thickness, nuclear pleomorphism, higher than 1/10 HPFs mitotic activity, or over 4% Ki67 index. The main difference is the neoplastic infiltration of the adjacent tissue (that requires specific immunohistochemical staining underlining the vascular plot to improve the diagnosis) [74]. Although presenting some of these shared features, both of our patients had vascular invasion within the capsule or the surrounding tissue, confirming the malignancy.

The American Joint Committee on Cancer (AJCC) eighth edition recently proposed a new staging classification for these tumors [7]. The atypical PA has been labeled as an “atypical parathyroid tumor” to reflect the uncertain malignant potential and emphasize the need for careful follow-up. However, we did not find any literature reports of atypical PA associated with SHPT.

Of PC patients, 10 to 30% have distant metastases and 6 to 30% have lymph node metastasis at diagnosis. Imaging (PET or whole-body MIBI scintigraphy) and biochemical surveillance (PTH and serum calcium) are needed [1,75].

The prognosis of these patients roughly depends on the success of surgery, as PTx is considered the only curative treatment [73]. PC are generally considered to be radioresistant; however, external beam radiotherapy (EBRT) is reserved for relapses as a palliative option. Chemotherapy and immunotherapy have now shown efficacy in treating local or distant metastases [1,72,73].

The mean disease-free interval between initial surgery and recurrence (approximately 50% of cases) is around 3 years, with much longer intervals also reported. Therefore, lifelong surveillance appears valid. The 5-year survival rates reach 93%, with a 10-year survival rate of approximately 67%, depending on initial surgery, age, initial biochemical remission, and the presence of metastasis [1,72]. Most of the SHPT patients with PC reported in the literature had a rather good evolution, despite the postoperative discovery and the absence of initial “en bloc” resection; some patients had favorable outcomes despite the evolution not being completed by radical re-intervention. Poor prognosis was conditioned by the presence of lung metastasis. One of our patients showed biochemical remission and absence of local or distant metastasis, despite the absence of “en bloc” resection, and the other one with bilateral PC had a suspicious renal mass that is yet to be investigated. It is possible that PC in SHPT has a more benign course and better outcomes compared to PC in PHPT: despite the malignant transformation that appears at the endpoint of the natural course of chronic PTH stimulation, various mechanisms, and interventions that interfere with the pathophysiology of the parathyroid gland (calcimimetics, vitamin D analogues, and increased FGF23) may contribute to a more indolent evolution. Moreover, parathyroids in SHPT mimic the changes undergone by PC in PHPT, making the presurgical diagnosis very difficult to achieve. The much lower prevalence of PC in SHPT compared to PHPT and its more indolent evolution with better prognosis strongly suggests that the genetic background for PHPT comes with an increased risk of parathyroid malignancy, not necessarily present in the patients with ESRD-related SHPT.

In conclusion, most features that raise the suspicion of PC in PHPT are part of the usual SHPT evolution in CKD and, thus, are not valid in SHPT. Still, most SHPT patients were refractory to medical therapy and had hypercalcemia accompanied by PTH values > 1000 pg/mL (including our second case), while those with multiple synchronous malignant lesions had a longer dialysis vintage (our first case here included) or very long-term immunosuppression (>10 years). The finding of a palpable neck mass accompanied by hypercalcemia, moderately elevated PTH values but resistant to calcimimetics or very high serum calcium (>13–14 mg/dL), and very high MIBI uptake or ultrasonographic features suggestive of malignancy (irregular margins, calcifications, thick capsule, and depth/width ratio > 1) in an ESRD patient in his 40s or 50s should raise concern and ideally be followed by alternative imaging techniques such as a CT scan.

PC in ESRD may be an incidental finding, or the end-step of the natural transformation of chronically stimulated parathyroids upon which other predisposing factors add (e.g., irradiation, immunosuppression, and genetic predisposition). Despite its incidental finding, most patients have favorable outcomes if distant metastases are absent, even in the absence of “en bloc” resection.

## Figures and Tables

**Table 1:** Biochemical data at diagnosis and after the surgery intervention for case 1.

Parameter	Normal Range	At Diagnosis	UCT	On Admission for Surgery	Time after Surgery			
					One Day	One Month	6 Months	9 Months
**PTH (pg/mL)**	15–65	1048	1136	1309	NA	270.2	590	491.7
**Ca (mg/dL)**	8.8–10.3	9.7	8.71	9.6	8.08	9.41	9.61	9.4
**P (mg/dL)**	2.5–4.8	7.2	5.78	8.72	NA	4.02	4.5	5.9
**ALP (UI/L)**	48–116	NA	NA	NA	NA	72	66	NA

ALP = alkaline phosphatase; Ca = albumin corrected calcium; NA = not assessed; P = phosphorus; PTH = parathormone; UCT = under conservatory treatment for 6 months.

**Table 2:** Biochemical data at diagnosis and after surgical intervention for case 2.

Parameter	Normal Range	On Admission for Surgery	Time after Surgery			
			2 Months	8 Months	10 Months	16 Months
**PTH (pg/mL)**	15–65	1911	793	534.05	889.05	585
**Ca (mg/dL)**	8.8–10.3	10.4	7.8	10.12	8.2	10.1
**P (mg/dL)**	2.5–4.8	3.2	2.25	3.3	3.33	5.2
**ALP(UI/L)**	48–116	161	161	NA	75	55

ALP = alkaline phosphatase, Ca = albumin corrected calcium; NA = not assessed; P = phosphorus; PTH = parathormone.

**Table 3:** Characteristics of parathyroid carcinoma cases associated with SHPT identified in the literature.

n^o^	Case	Age, Sex	HD/KT y	Ca mg/dL	PTH pg/mL	CTt	L/W cm/g	Risk Factors	Local Invasion/Metastasis	Histology/Surgical Approach	Outcome Follow-Up Months
1	Berland et al. 1982 [13]	62, F	3 -	9.2	1820	NA	NA	-	-	2 PC Subtotal PTx + arm implant	Good
2	Anderson et al. 1983 [14] *	44, F	NA	↑	NA	NA	NA	-	NA	NA	Bad
3	Ireland et al. 1985 [21]	34, M	5 -	12.3	1043	NA	2 NA	Neck Rx (laryngeal cancer)	lung	1 PC Total PTx with forearm autograft Reintervention: resection of autograft due to hypercalcemia	Bad
4	Sherlock et al. 1985 [12]	42, F	7 -	12.9	>10,000 PTH-C	NA	4 NA	-	local vessels, thyroid	1 PC Subtotal PTx + forearm implant	Good hPTH
5	Krishna et al. 1989 [22]	64, F	NA	11	>10,000 PTH-C	P binders	NA	-	thyroid	2 PC Total PTx	Good—36 m
6	Kodama et al. 1989 [23]	53, F	7 -	11.1	121.4	NA	3 NA	-	-	1 PC Subtotal PTx	Good
7	Iwamoto et al. 1989 [24]	46, M	10 -	9.6	24,200 PTH-C	D analogues	NA 3.3	-	recurrent nerve	1 PC Total PTx	NA
8	Iwamoto et al. 1989 [24]	55, F	5.5 -	9.4	98,300 PTH-C	D analogues	1 NA	-	thyroid	1 PC Subtotal PTx + forearm implant	NA
9	Rademaker et al. 1990 [25]	46, F	3 -	12.2	723	D analogues, P binders	3.5 NA	-	thyroid	1PC Total PTx Reintervention: subtotal thyroidectomy	Good—84 m hPTH
10	Rademaker et al. 1990 [25]	52, F	2 -	12.6	523	D analogues, P binders	2 NA	-	capsular invasion	1 PC Neck dissection on the side of the tumor	Good—48 m
11	Tominaga et al. 1995 [26]	46, F	20 -	6.1	956	NA	NA 10	-	thyroid gland, local lymph nodes, lung metastasis	Misinterpreted as a benign lesion: Total PTx; Reintervention: resection of 10 parathyroid nodules (2 y after) + total thyroidectomy: Reintervention: lung metastasis resection (classified as PC after metastasis)	Ameliorated- persistent, but stable SHPT
12	Miki et al. 1996 [27]	40, F	4.5 -	7.8	62, 000 PTH-C	D analogues	NA 2.7	-	multiple, bilateral, recurrent lung metastasis	1 PC Total PTx + forearm implant Reintervention: right lobectomy of the thyroid gland (papillary carcinoma), partial left pulmonary resection + right pulmonary resection for lung metastasis (2 y years after).	Poor—persistent SHPT, lung and neck masses
13	Liou et al. 1996 * [14]	64, M	0 -	14.7	↑	-	NA	-	-	1 PC Subtotal PTx	Good—“Hungry bones”
14	Tseng et al. 1998 [28]	20, F	5 -	12.7	1143	D analogues	NA 6.2	-	lung metastasis	4 PH Subtotal PTx Reintervention: 1 y after: + 4th parathyroid and thymus + resection of left pulmonary lobe nodule (for lung metastasis;	Bad—hypercalcemia, recurrent lung metastasis, exitus
15	Takami et al. 1998 [29]	55, F	10 -	10.9	5080	NA	2.4 NA	-	capsular and vascular invasion; sternothyroid m, esophagus, thyroid	1 PC Total PTx + Right lobectomy of thyroid + lymphadenectomy + sternothyroid and adventitia of esophagus + forearm implant	Good—4 m
16	Jayawardene et al. 2000 [30]	74, F	3 -	12.06	1303	-	NA	-	vascular invasion	1 PC Total PTx	Good—48 m hypocalcemia
17	Kuji et al. 2000 * [14]	51, M	22 -	↑	↑	NA	NA	-	-	1 PC Total PTx + forearm implant	Good
18	Zivaljevic et al. 2002 [31]	69, M	6 -	10.82	1901	NA	5 NA		thyroid, sternothyroid m.	1 PC Total PTx + thyroid lobe+ sternothyroid m resection	Good—7 m hPTH
19	Srouji et al. 2004 [32]	27, M	- 10	11.2	1405	D analogues	NA	10 y IS for KT	thyroid, thymus; mediastinum; parathyromatosis	1 PC Total PTx (misinterpreted as benign) Reintervention 1 y afterradical neck + mediastinal dissection for parathyromatosis	Good—9 m
20	Khan et al. 2004 [33]	33, M	8 -	10.6	597	D analogues	NA 11	-	lung, soft tissues adjacent to the scapulae	1 PC—after reexamination, Subtotal PTx + ½ preserved parathyroid;	Poor—palliative care; multiple fractures
21	Bossola et al. 2005 [34]	52, F	3 -	12.4	1366	NA	NA	-	-	1 PC Subtotal PTx	Good
22	Babar-Craig et al. 2005 [35]	55, M	NA	↑	↑	NA	NA	-	-	1 PC Total PTx	NA
23	Falvo et al. 2005 [14] *	61, M	18 -	↑	↑	NA	NA	-	-	2 PC Total PTx + total thyroidectomy	Good
24	Tkaczyk et al. 2007 [36]	55, M	0 -	11.6	2807	CaR antag, D analogues	2.7 NA	-	mediastinal adipose tissue	1 PC Total PTx Reintervention: mediastinum ectopic parathyroidectomy and “en bloc” lymph node resection	Good—7 m “Hungry bones”
25	Diaconescu et al. 2011 [37]	48, M	13 -	10.42	710	NA	3 NA	-	-	1 intrathyroidal PC misinterpreted as a thyroid nodule Total PTx + thyroidectomy	Good—hPTH
26	Nasrallah et al. 2014 [14] *	53, M	NA	11.1	324	NA	NA	-	laryngeal nerve branch	1 PC Total PTx	Good
27	Kim et al. 2016 [38]	57, M	11 2	10.6	1287	cinacalcet	1.7 NA	2y IS for KT	capsular invasion	1 PC Subtotal PTx (3 + 1/2)	Good—6 m
28	Pappa et al. 2017 [39]	45, M	4 -	10.6	1422	cinacalcet	3 NA	-	vascular and capsular invasion	1 PC Total PTx	Good—36 m
29	Curto et al. 2019 [40]	59, F	NA 40	14	1544	NA	1 NA	40y IS for KT	lung metastasis, capsule invasion, infiltration of m, fat	4 PC First intervention: Lung lobectomy for a suspicious lesion (histological PC metastasis) Reintervention: “en bloc” resection	Good
30	Shen et al. 2019 [41]	70, M	2 -	15.11	197	cinacalcet calcitriol	2.5 NA		-	ectopic mediastinal PC: video-assisted thoracoscopic guided removal	Good
31	Won et al. 2019 [42]	46, M	8 Rej. KT	9.8	1399	paricalcitol cinacalcet, sevelamer	2.5 NA	IS for KT	muscle and vascular invasion	1 autograft PC Total PTx + SCM autograft	Good—5 m
32	Cappellacci et al. 2020 [43]	51, M	15 -	10.7	2582	sevelamer	2.5 NA	-	vascular and capsular invasion	1 PC Total PTx	Good—22 m “Hungry bones”
33	Malipedda et al. 2020 [44]	53, M	5 -	12.5	3360	NA	1.3 NA	-	vascular invasion	1 PC Subtotal PTx + ½ forearm implant	Good
34	Kada et al. 2021 [45]	48, F	15 -	8.9	830	NA	2 NA	-	esophageal mucosa muscle plate	1 PC Total PTx + bilateral peritracheal lymph node dissections	Good—100 m
35	Chen et al. 2022 [46]	49, M	0 -	High	1483	NA	1.8 NA	-	-	1 PC Total PTx + posterior median sternotomy	Good
36	Radu et al. 2023 [15]	35, M	3 -	11.6	804	NA	2 NA	-	capsular and vascular invasion	1 PC Total PTx	Good—48 m
37	Radu et al. 2023 [15]	55, F	5 -	13.2	1283	NA	2 NA		-	1 PC Total PTx	Dis. free—60 m Exitus: heart dis.
38	Ryang et al. 2022 [47]	54, M	13 -	10.6	1144	sevelamer, paricalcitol cinacalcet	2.2, 2.2, 1.5	-	capsular invasion	3 PC Total PTx + forearm autograft	Good—hypocalcemia
39	Yokoyama et al. 2023 [14]	54, F	14 -	11.4	1007	maxacalcitol	5.9 NA	-	thyroid, recurrent nerve	1 PC Total PTx + ” en bloc” resection + arm autograft	Good—4 m
40	Yang et al. 2023 [20]	46, F	4 -	11.8	1672	none	2.5 NA	-	parathyromatosis	1 PC and parathyromatosis Total PTx 5 years before Reintervention: neck exploration and removal of PC and parathyromatosis	Good—8 m
41	Salimkhanov et al. 2023 [48]	48, F	3 -	12.7	3556	cinacalcet	3.8 NA	-	parathyromatosis thyroid	multifocal intrathyroidal PC after total PTx Reintervention: thyroidectomy	Sagliker syndrome Ameliorated
42	Mahmood Bin et al. 2023 [49]	53, M	0 -	12.4	1347	NA	NA	-	-	ectopic mediastinal PC: video-assisted thoracoscopic guided removal of the parathyroid tissue	Good

CasR = calcium sensing receptors; CTt = conservatory treatment; D = vitamin D; HD = hemodialysis; hPTH = hypoparathyroidism; IS= immunosuppression; KT = kidney transplant; L = length (cm) of the involved parathyroid; M = male; m = muscle; NA = not available; P = phosphate; PC = parathyroid carcinoma; PTx = parathyroidectomy; PTH-C = C terminal parathormone; Rx = radiotherapy; SPTH = secondary hyperparathyroidism; W = weight of involved parathyroid (g); * Data collected from previous reviews as stated.

## Data Availability

The original contributions presented in the study are included in the article. Further inquiries can be directed to the corresponding author.
